# Date Palm Thorn Injury: A Literature Review and a Case Study of Extensive Hand Haematoma

**DOI:** 10.7759/cureus.13252

**Published:** 2021-02-10

**Authors:** Saif Badran, Mazin Mohammed, Iqbal Wani, Atalla Hammouda, Salim Al-lahham

**Affiliations:** 1 Department of Plastic Surgery, Hamad Medical Corporation, Doha, QAT; 2 Department of Population Medicine, College of Medicine, Qatar University (QU) Health, Qatar University, Doha, QAT

**Keywords:** date palm, thorn, bleeding, synovitis, granuloma, phoenix dactylifera

## Abstract

Date palm thorn injuries require a high level of clinical suspicion and careful management as they can lead to severe complications, such as tissue inflammation, synovitis, and extensive haematoma. Although it is associated with serious injuries, this type of injury is often underdiagnosed and is not sufficiently reported in the literature. We describe a case report of an 18-year-old male patient who presented with significant progressive swelling of the right hand that extended to the distal third of the forearm, having received a date thorn injury the day before. The patient underwent multiple incisions and hand fasciotomy to reduce the haematoma and relieve compartment pressure in his hand.

## Introduction

Date palm (*Phoenix dactylifera*) is a flowering plant species prevalent in the Middle East, the United States, and New Zealand [1]. Although they are associated with serious injuries from thorns, this type of injury remains underdiagnosed and is not extensively reported in the literature for numerous reasons. These include the tendency of children to be injured by an array of objects and the lack of a clear history of the injury, the ability of minute thorn particles to break off inside the tissue and be easily overlooked, and the radiolucent characteristics of the thorn pieces on X-ray film [2,3].

Making a clinical diagnosis of a date palm thorn injury can be challenging for the treating physician, especially when the injury history is unclear and the signs are nonspecific. Plain X-ray films cannot show the thorn pieces as they are radiolucent, but they help detect bone and joint changes, such as effusion and a periosteal reaction, which can be late presentations of this disease [3]. Ultrasound is more helpful in localising the thorn particles and soft tissue changes but requires a skilled operator [4]. Computerised tomography has greater sensitivity in identifying foreign particles, such as thorn pieces, than magnetic resonance imaging; however, the latter is more useful in detecting associated inflammation in the soft tissue [5]. A general laboratory test of the patient’s serum or synovial fluids after joint aspiration typically reveals a negative or nonspecific inflammatory change [6,7].

Thus, date palm thorn injuries require high clinical suspicion and careful management as they can involve serious complications that depend on the location and depth of the injury, the type of soft tissue involved and the patient’s general health condition [3].

## Case presentation

An 18-year-old epileptic male patient, controlled with levetiracetam 500 mg twice daily and valproic acid 750 mg twice daily, with a history of right reflux nephropathy since childhood (stable chronic kidney disease managed with regular follow-ups), presented to the accident and emergency department complaining of slight swelling and mild pain in his right hand since one hour. He had accidentally hit his hand against a palm date thorn, which had pierced his skin; however, he was able to remove it immediately. 

On examination, mild inflammatory swelling was observed on the dorsum of the right hand, together with a small puncture wound. Remnants of the thorn were not identified using a plain X-ray; the only finding was minimal soft tissue swelling. The patient was discharged with a pressure dressing, an analgesic, and an antibiotic (amoxicillin/clavulanate [Augmentin®], 1 g, every 12 hours for a one-week duration). He was advised to elevate his hand as far as possible. 

**Figure 1:**
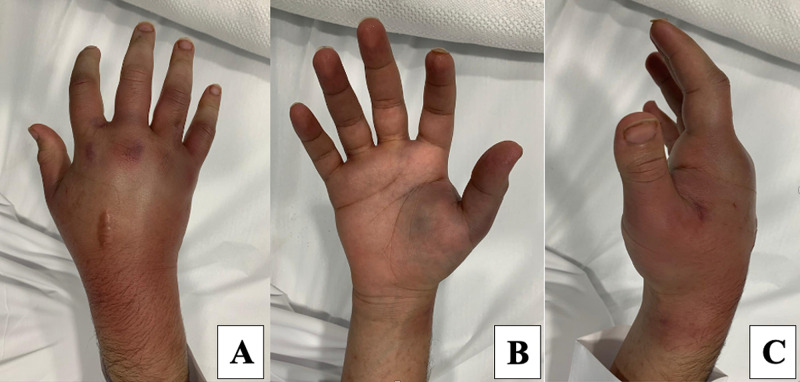
Right hand examination showing extensive hand swelling extending dorsally from the metacarpal joints to the distal forearm, associated with redness and ecchymosis. A: posterior view, B: anterior view, C: lateral view.

He presented the next day again with significant progressive pain and hand swelling, which extended to the distal third of the forearm (Figure 1) and had started a few hours. The patient did not report any systematic symptoms of infection or additional hand trauma. His examination showed tense hand swelling extending dorsally from the metacarpal joints to the distal forearm, associated with redness, ecchymosis, heat, positive fluctuations, and decreased mobility in his fingers.

**Figure 2:**
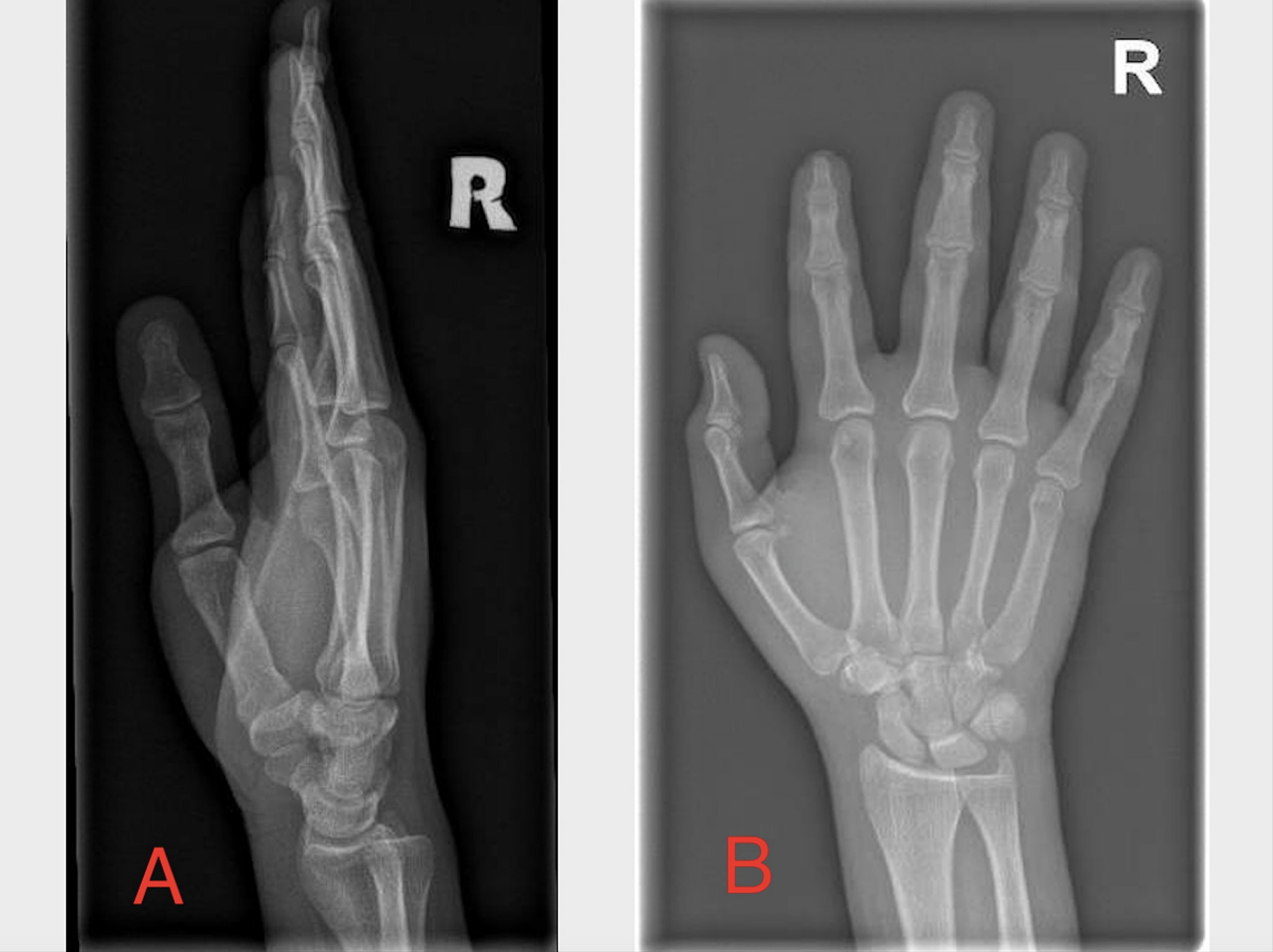
Right hand X-ray showing soft tissue swelling, no foreign body noted. A: lateral view, B: anteroposterior view

A second X-ray was performed. Again, only soft tissue swelling was identified; foreign bodies, gas bubbles, and fractures were not observed (Figure 2). The laboratory investigation included a complete blood count, complete metabolic profile, coagulation profile, urine analysis, and a C-reactive protein test. Other than a marginally high white blood count (10.1 × 10^3/uL), no abnormalities were observed. Differential diagnosis included hand abscess, haematoma, and tenosynovitis. The latter was excluded due to the dorsal location of the injury and the absence of Kanavel signs. 

**Figure 3:**
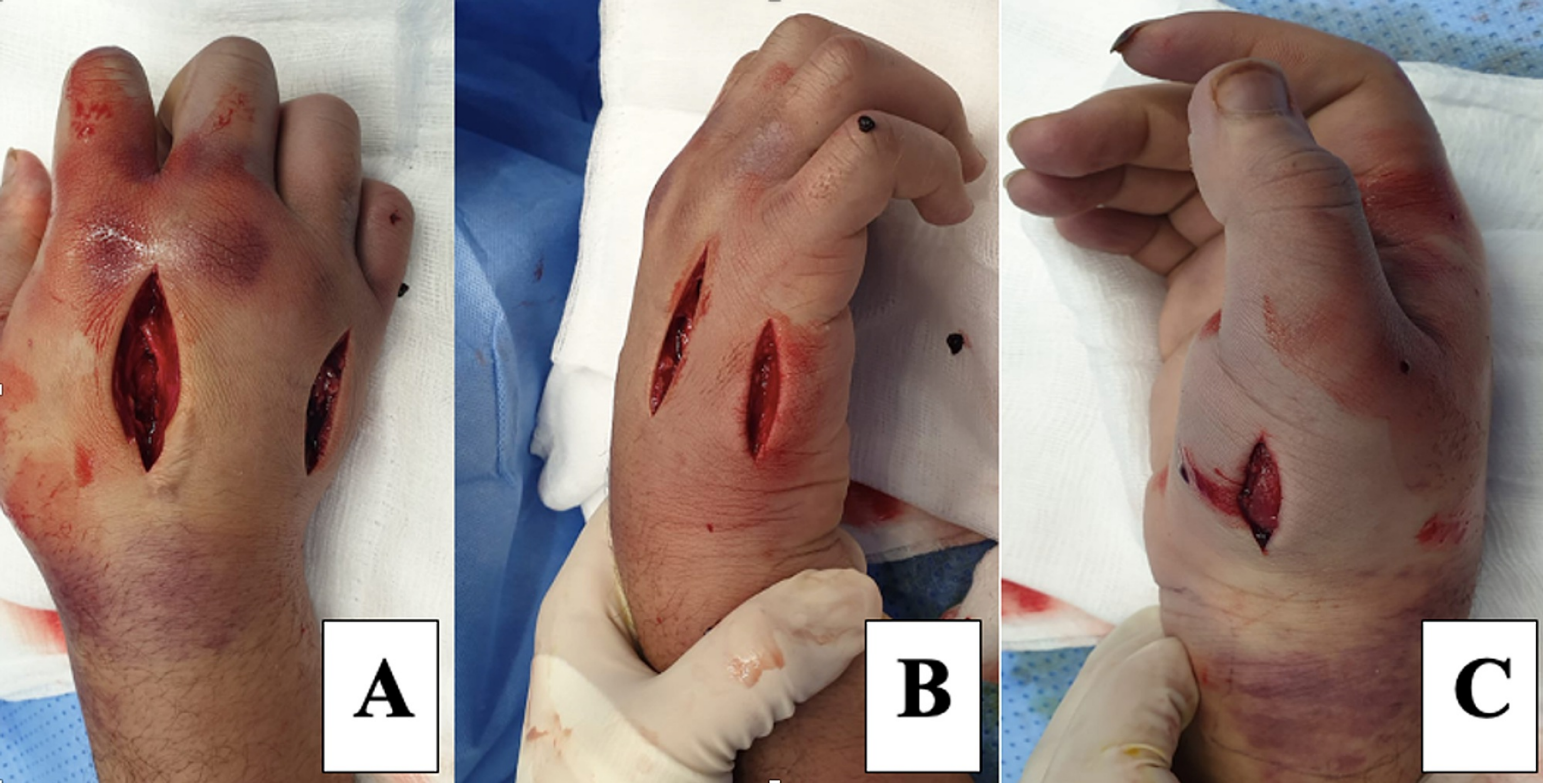
Intraoperative photos of the right hand, multiple dorsal and side incisions along with hand fasciotomy. A: dorsal view, B: medial view, C: lateral view.

The patient was started on an analgesic and an intravenous antibiotic (amoxicillin/clavulanate [Augmentin®], 1.2 g, every eight hours), the hand was elevated, and he was booked for surgery within a few hours. Intraoperatively, multiple dorsal and lateral incisions, together with hand fasciotomy, were performed to reduce the swelling and pain associated with the haematoma and dorsal compartment pressure (Figure 3). No thorn remnant was noted. The wound edges were loosely approximated using nonabsorbable sutures, and a light dressing was applied. 

The patient was discharged after two days with an oral analgesic, anti-inflammatory medication, and an antibiotic (amoxicillin/clavulanate [Augmentin®] 1 g, every 12 hours, one-week duration). He was asked to change the dressing daily. 

**Figure 4:**
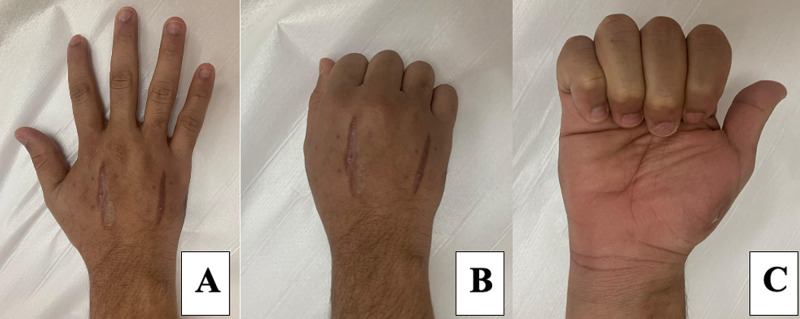
Normal hand range of motion one month after surgery. A: fingers extended and abducted (posterior view), B: fist formation (posterior view), C: fingers flexed (anterior view).

The patient was followed-up at the clinic after six weeks. The examination showed resolution of the swelling, normal range of motion in the hand, and multiple healed scars (Figure 4). He was referred to an occupational therapist and discharged.

## Discussion

Date palm thorn injuries demand a high level of clinical suspicion by the treating physician as they fall within a specific category of thorn injury; the thorns have specific physical characteristics that result in the penetration of deep soft tissue and bone, which can eventually manifest as serious complications. 

Soft tissue inflammation is a common outcome of date thorn injuries, often leading to pus formation due to secondary infection by* Pantoea agglomerans*, a Gram-negative aerobic bacillus in the Enterobacteriaceae family, which results in substantial inflammation [8]. Injuries near the joints can result in monoarticular synovitis that can be resistant to conservative and medical treatments [1,9] and often requires therapeutic arthroscopic surgery with extensive synovectomy as limited synovectomy is associated with a high risk of incomplete resolution [3]. It is vitally important to locate the anatomical location and features of the injury by radiology before the patient is scheduled for arthroscopy as the tiny pieces of thorn might not be visible during the procedure due to associated soft tissue inflammation and swelling [5].

The sharpness of date thorns means that the injuries can involve the bone in certain cases, resulting in a nonspecific granuloma as a reaction to the foreign body [4,7]. Rarely, the piece of thorn within the bone is surrounded by a fibrotic capsule and is characterised by an osteolytic reaction that can appear as a pseudotumour upon imaging, which makes a diagnosis more challenging [1].

This case report highlights the potential haemorrhagic complications associated with date thorn injuries that can lead to significant morbidity. In addition, the degree to which a small thorn prick can result in extensive haematoma and swelling is often underestimated. Alan et al. reported on a case of a palm date thorn injury to the left calf of a 49-year-old otherwise clinically healthy gentleman; it resulted in significant haematoma associated with transient haematuria [10]. Although vascular haematoma is a differential diagnosis, however, the site of injury in our case, the dorsum of the hand, is not known to be a large anatomical vessel required for the formation of an extensive haematoma. It might be clinically helpful in future to test the thorn for potential toxins, although this might be challenging and impractical in certain situations.

## Conclusions

Date palm (*Phoenix dactylifera*) thorn injuries are common in certain parts of the world. They require a high level of clinical suspicion and careful management by the treating physician as they can lead to serious complications, such as severe tissue inflammation, synovitis and extensive haematoma. Making an accurate diagnosis can be challenging, especially in children without a clear history of the injury. Ultrasound imaging can be helpful to locate the retained thorn particles.
